# Artificial Intelligence–Mediated Discharge Document for Accessible Health Care (AIM-HEALTH): Protocol for a Prospective, Observational, Noninterventional Study

**DOI:** 10.2196/95782

**Published:** 2026-07-03

**Authors:** Nicola Pelizzari, Mattia Savardi, Luca Arrigoni, Marco Gregori, Adelaide Conti, Martina Tedesco, Elena Tratta, Elisabetta Ceretti, Loredana Covolo, Maria Visconti, Marianna Adamo, Aldo Roccaro, Enrico Vizzardi, Alberto Signoroni, Federico Alberici, Carlo Mario Lombardi, Umberto Gelatti

**Affiliations:** 1 Department of Medical and Surgical Specialties, Radiological Sciences, and Public Health University of Brescia Brescia, Lombardy Italy; 2 Cardiology Unit Azienda Socio Sanitaria Territoriale degli Spedali Civili di Brescia Brescia, Lombardy Italy; 3 Nephrology Unit Azienda Socio Sanitaria Territoriale degli Spedali Civili di Brescia Brescia, Lombardy Italy; 4 Forensic Medicine Unit Azienda Socio Sanitaria Territoriale degli Spedali Civili di Brescia Brescia, Lombardy Italy; 5 Centre of Bioethics Research University of Brescia Brescia, Lombardy Italy; 6 Clinical Ethics Consultation Service Azienda Socio Sanitaria Territoriale degli Spedali Civili di Brescia Brescia, Lombardy Italy; 7 Clinical Trial Center Azienda Socio Sanitaria Territoriale degli Spedali Civili di Brescia Brescia, Lombardy Italy

**Keywords:** hospital-to-home transitions, health literacy, on-premise large language models, AI-driven public health communication, discharge communication, artificial intelligence

## Abstract

**Background:**

Hospital discharge reports (HDRs) support continuity of care; yet, their specialized terminology may hinder patient understanding and postdischarge self-management, particularly among individuals with limited health literacy (HL).

**Objective:**

AIM-HEALTH (Artificial Intelligence–Mediated Discharge Document for Accessible Healthcare) aims to develop and evaluate a clinician-validated, artificial intelligence (AI)–powered supplementary discharge document (SDD) to support comprehension of HDR content, tailored to patients’ HL levels.

**Methods:**

This prospective, observational, noninterventional study will enroll 200 adults from the nephrology and cardiology units at Azienda Socio-Sanitaria Territoriale Spedali Civili hospital. Following written informed consent, participants’ HL will be assessed and combined with HDR structured data and an audio-recorded discharge interview to generate two outputs using locally deployed agentic AIs powered by an open-weight large language model: (1) an HL-tailored SDD for patient use and (2) a clinical informational performance report (CIPR) highlighting omissions and inconsistencies between the HDR and the discharge interview to support clinical safety. Clinicians will validate the SDD using a QUEST (quality, understanding, expression style, safety, and trust)–informed tool assessing accuracy, completeness, clarity, utility, and safety domains. All outputs will undergo clinical assessment; only suitable outputs will be retained, while corrections and unsuitable outputs will be used to iteratively refine the system. Patients will assess SDD perceived accessibility, comprehensibility, usefulness, and engagement. Data processing follows on-premise, Data Protection Impact Assessment–defined safeguards (data minimization, pseudonymization, and security or incident management).

**Results:**

Expected results include (1) technical feasibility and workflow indicators (completion and SDD withholding rates); (2) clinician-rated accuracy, appropriateness, completeness, and safety of SDDs; (3) perceived utility of the clinical informational performance report for identifying omissions and inconsistencies between HDRs and discharge interviews; (4) patient-reported accessibility, comprehensibility, usefulness, and engagement at ~1-month follow-up; and (5) associations between SDD readability index scores and perceived comprehensibility. This study is approved by the local institutional review board (NP 6761-62, February 24, 2026). Data collection commenced on May 29, 2026.

**Conclusions:**

AIM-HEALTH is intended to generate preliminary feasibility, safety, and acceptability evidence on the integration of an AI-generated, HL-adapted SDD into routine workflows, particularly for patients with chronic conditions. The findings are intended to inform the design of future comparative studies addressing the potential role of such tools in postdischarge communication, under on-premise data protection and mandatory clinician validation.

**International Registered Report Identifier (IRRID):**

PRR1-10.2196/95782

## Introduction

Hospital discharge reports (HDRs) are core clinical documents that synthesize inpatient stay and formally convey diagnoses, prescribed therapies, follow-up plans, and lifestyle recommendations, thereby ensuring informational continuity of care across hospital services, patients, and community-based providers [1-3]. However, extensive evidence shows that HDRs are often written in complex medical language, characterized by dense terminology and intricate syntactic structures that many patients, especially those with limited health literacy (HL), find difficult to understand [3]. This mismatch between document complexity and patients’ HL has been linked to multiple adverse outcomes, including limited understanding of written discharge instructions, difficulties in following prescribed therapies, and a higher likelihood of complications and potentially avoidable hospital readmissions [4]. Evidence across several inpatient settings shows that misunderstanding of discharge instructions is widespread, with most patients failing to fully understand at least 1 critical element in the HDRs, particularly medications and return-to-care indications [5,6]. In Italy, studies highlight substantial challenges in both general literacy and HL: the Health Literacy European Survey 2019–2021 indicates that 23% of adults have inadequate and 35% problematic HL, while Organisation for Economic Co-operation and Development data estimate that around 28% of Italian people aged 16-65 years are functionally illiterate [7,8]. These figures suggest that a large proportion of patients are likely to experience systematic difficulties in processing standard HDRs, particularly when managing chronic and multimorbid conditions that require complex self-care [3,4].

At the same time, clinicians work under increasing administrative and documentation burdens and must comply with stringent clinical and legal standards, which limits the time available to tailor discharge communication to individual patients’ HL levels and informational needs [9]. As a result, HDRs often remain predominantly clinician-centered, with written materials optimized for professional and legal requirements rather than for patient comprehension and usability [10]. This structural imbalance disproportionately affects patients with lower HL, who are precisely those at greatest risk of misunderstanding critical information on medication, symptom monitoring, and follow-up, and who may therefore face higher probabilities of unsafe postdischarge trajectories [11,12]. Improving the clarity, accessibility, and usability of HDRs is thus a key priority for promoting patient autonomy, adherence, and safety in the transition from hospital to home, particularly in the context of chronic diseases [13,14].

Artificial intelligence (AI), and in particular, generative large language models (LLMs), has recently emerged as a promising resource to support the simplification and personalization of clinical documentation, including discharge-related texts [15-23]. Studies in various clinical domains indicate that AI-generated summaries and reports can be perceived as clearer and more concise than traditional clinician-authored documents while maintaining comparable clinical accuracy and completeness [16-23]. Patients and clinicians frequently rate AI-assisted texts as more readable and user-friendly, and generative systems have shown the ability to reproduce most key clinical items, such as medication instructions and follow-up recommendations [24-29]. In parallel, AI-based documentation tools are being explored as a means to reduce clinicians’ administrative burden, mitigate variability in document quality, and support more consistent communication practices [26-30]. Nonetheless, existing work remains fragmented, and the implications of using AI-generated, patient-facing materials in routine hospital discharge processes are not yet fully understood [29-31].

Several important gaps persist in this emerging field. First, most published studies are proof-of-concept or pilot evaluations conducted in English-language settings and do not address morphologically rich languages such as Italian or the organizational and regulatory specificities of the Italian National Health Service [16-30]. Second, many evaluations focus on single dimensions, for example, perceived clarity or basic accuracy, without combining clinical, linguistic, and experiential metrics within a shared framework that can robustly capture the quality and safety of AI-generated documents. Third, HL is rarely incorporated as an explicit personalization variable: AI-generated texts are typically not adapted to patients’ measured HL levels, despite the well-documented role of HL in shaping comprehension, self-management, and outcomes in chronic disease [20]. Finally, ethical and legal considerations, including transparency, bias, accountability, and data protection, are not consistently integrated into the design, governance, and evaluation of AI-mediated communication tools, raising concerns about their trustworthy deployment in real-world care [24,30,32].

The AIM-HEALTH (Artificial Intelligence–Mediated Discharge Document for Accessible Healthcare) project has been conceived to address these gaps at the intersection of health communication, applied linguistics, clinical informatics, and AI ethics in the Italian hospital context. Focusing on adult patients with chronic conditions, AIM-HEALTH targets hospital HDRs in nephrology and cardiology units at a large Italian university hospital, where clear written information is crucial to safe and effective postdischarge management [2,6]. Within this setting, the project introduces the concept of a supplementary discharge document (SDD), generated with AI and designed as a patient-facing complement to the standard HDR, with a specific emphasis on linguistic accessibility and sensitivity to patients’ HL profiles [18,21,22,27-29]. The primary objective of this study is to assess the clinical quality and safety of the AI-generated SDD, as evaluated by physicians on the basis of the source documents, prior to any patient-facing use. Secondary objectives are (1) to characterize patient-reported accessibility, comprehensibility, usefulness, and engagement with the SDD; (2) to explore clinicians’ perceived utility, in clinical practice, of an automated tool that highlights potential omissions and inconsistencies between the written HDR and the discharge interview; (3) to assess the feasibility and acceptability of integrating an HL-sensitive, AI-mediated SDD into routine clinical workflows; and (4) to describe the readability of the AI-generated SDD and of the corresponding HDR. Exploratory objectives are to examine potential associations between linguistic features of the SDD and patient-reported outcomes and to describe possible differences in the use and perception of the SDD across clinical units and across HL profiles. These analyses are intended to be hypothesis-generating and to inform the design of future comparative studies. By focusing on these questions, AIM-HEALTH seeks to generate evidence on the potential and limitations of HL-sensitive, AI-mediated discharge communication in an Italian public hospital setting and to inform future models of responsible, patient-centered AI use in clinical communication.

## Methods

### Study Design, Setting, and Participant Recruitment

This study is a prospective, observational, noninterventional study designed to evaluate the use of AI in the generation of SDDs intended to support the HDRs. The study adopts a nonrandomized, open-label design, as all participants receive the same procedures, and no allocation to control or comparison groups is planned. The study is conducted at Azienda Socio-Sanitaria Territoriale (ASST) Spedali Civili di Brescia (Italy), a large tertiary-level public teaching hospital and referral center for the Lombardy region. The hospital is part of the Italian National Health Service and is formally affiliated with Università di Brescia, supporting integrated activities in clinical care, research, and medical training. ASST Spedali Civili di Brescia provides highly specialized inpatient and outpatient services across medical, surgical, and subspecialty areas and manages a high volume of patients with complex acute and chronic conditions. Within this institutional context, the study is carried out in the nephrology unit and the cardiology unit. These clinical settings were selected due to the high communicative demands associated with the postdischarge management of chronic nephrological and cardiovascular conditions, where clear, accurate, and accessible discharge information is essential to ensure continuity of care, medication adherence, and effective self-management after hospitalization.

The planned sample consists of 200 adult patients, equally distributed between the nephrology and cardiology units (n=100 per unit). The sample size was determined on pragmatic grounds informed by published guidance on pilot and feasibility study design, which suggests that samples of this magnitude may be reasonable to characterize feasibility and acceptability and to inform estimates of key design parameters of complex interventions prior to comparative evaluation [33-35]. The planned sample is also larger than those reported in several published pilot evaluations of AI-generated discharge documents, which have often involved smaller samples [28]. As an indicative reference, under standard assumptions and using methods appropriate for binomial proportions, an observed proportion of 50% in a sample of 200 would correspond to a 95% CI of approximately ±7 percentage points; the corresponding interval for 100 would be approximately ±10 percentage points, and intervals around less frequent proportions (eg, 10%-20%) would tend to be narrower. With respect to associations between continuous linguistic, clinical, and patient-reported variables, a sample of 200 may allow detection of correlations on the order of *r*≈0.20 with reasonable sensitivity at α=.05 (2-sided), although actual sensitivity will depend on the empirical distributions and missingness observed during the study. Subgroup comparisons (eg, across HL categories or between clinical units) and analyses involving HL or fine-grained linguistic indicators are prespecified as exploratory and hypothesis-generating; the study is not designed to detect small effects within subgroups or to support inferences about efficacy. Findings are intended to inform the design of future comparative or multicenter studies. Patients are identified through a systematic review of discharge lists by clinical staff in the participating units to ensure representativeness of the target population. Eligible patients receive detailed information about the study and are asked to provide written informed consent prior to participation. Inclusion criteria comprise age ≥18 years, adequate reading and comprehension of the Italian language, ability to comprehend and sign informed consent, discharge from one of the participating units, diagnosis of a chronic condition requiring ongoing follow-up, and clinical stability at the time of discharge. Exclusion criteria include age <18 years, diagnosed cognitive impairment or significant language barriers preventing informed consent, severe comorbidities or clinical instability requiring continued inpatient care, participation in other clinical studies that could interfere with the study objectives, institutionalization limiting autonomous management of health information, and explicit refusal to participate. Patient eligibility and data quality are monitored throughout the enrollment process to ensure continued adherence to inclusion and exclusion criteria and consistency with study objectives. Periodic procedural checks are conducted upon completion of approximately 30%, 60%, and 100% of the planned reports within each participating unit, with the aim of verifying recruitment procedures, data completeness, and protocol compliance.

### Data Collection

After preliminary eligibility screening, eligible patients are informed about the study objectives and procedures during an individual interview. Participants are provided with clear study information and are given the opportunity to read the information sheet, ask questions, and receive clarifications before providing written informed consent. After providing informed consent, participants complete the Italian version of the 16-item European Health Literacy Survey Questionnaire, a translated and psychometrically validated instrument for assessing HL in the general population. In addition, participants complete the 3-item brief HL screen, a short validated screening tool assessing difficulties in understanding written health information, the need for assistance in reading medical materials, and confidence in completing medical forms [36]. The resulting HL score is recorded as a core study variable and is explicitly used as an input to the AI system to guide the linguistic complexity, structure, and informational density of the generated SDD. For each enrolled participant, the discharging physician produces the HDR according to routine clinical practice and institutional standards. In parallel, the clinician-patient discharge interview routinely conducted at the time of discharge is audio-recorded using a dedicated digital device as part of the study procedures. Audio recording is limited to the discharge encounter and is performed solely for the purposes of the study. Recorded discharge interviews are transcribed using an automated speech-to-text system based on the Whisper large-v3 model [37]. The resulting transcripts are used in conjunction with the HDR as primary source documents for the AI system. Together, the HDR and the transcript of the discharge interview constitute the inputs used both for the generation of the SDD and for the production of the clinical informational performance report (CIPR), enabling systematic comparison between written discharge documentation and transcribed verbal communication within the study workflow.

### AI System Architecture and Document Generation Workflow

**Figure 1:**
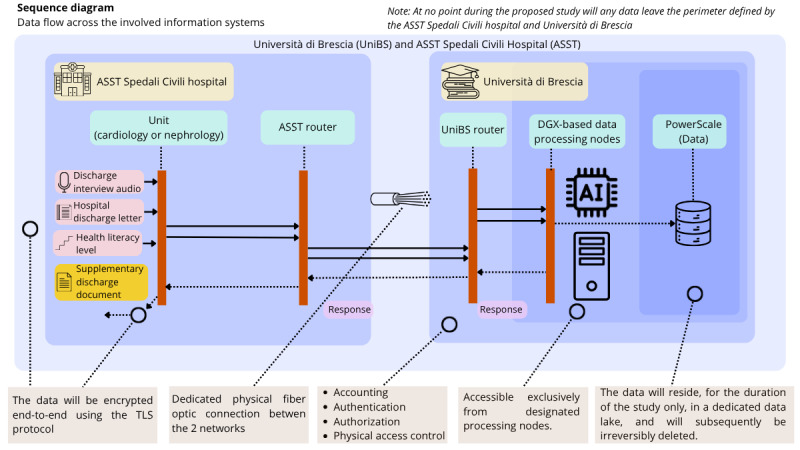
Data flow diagram. AI: artificial intelligence; ASST: Azienda Socio-Sanitaria Territoriale; DGX: Deep GPU Xceleration; TLS: Transport Layer Security.

The study uses a multiagent AI system based on LLMs to generate SDDs intended to complement the HDR. For each enrolled participant, the system integrates 3 inputs: structured information extracted from the HDR, the transcript of the audio-recorded discharge interview, and the participant’s HL score. These inputs are jointly processed to generate an HL-adapted SDD designed to support accessibility and comprehension of discharge information. The AI system is hosted entirely on local institutional high-performance computing infrastructure (NVIDIA DGX A100 graphics processing unit cluster) and is based on open-source LLMs, including Gemma 3 27B and Llama 2 70B, or derived versions. All processing is performed on-premise within institutional infrastructure, and no patient data are transferred to external cloud services. The system relies on pretrained models and does not involve model retraining using study data (Figure 1). During the study, patient data are not stored within the AI models and are not used for model retraining.

Document generation is implemented through a multiagent architecture comprising specialized agents responsible for data acquisition and structuring, analysis of clinical content through natural language processing, and synthesis and reformulation of information into an HL-adapted SDD. An orchestration mechanism coordinates agent interactions and manages the generation sequence to support coherence, accuracy, and reduction of informational complexity across outputs. Prior to full-scale enrollment, a dedicated technical development and validation phase is conducted. During this phase, a pilot dataset of 20 cases, with equal representation from the nephrology and cardiology units, is used to refine system prompts, agent coordination, and workflow parameters and to evaluate alternative model variants and system configurations, with the objective of optimizing output coherence, completeness, and alignment with clinical documentation standards. Refinement activities are limited to system-level configuration, prompt engineering, and orchestration logic, and do not involve modification of model weights. For each participant, the workflow produces 2 outputs. The first output is the SDD intended for patient use. The second output is the CIPR, designed to support clinical activities by checking omissions and inconsistencies between the HDR and the discharge interview and by highlighting critical information requiring clarification or reinforcement within the discharge process. The report also includes an assessment of potential discriminatory content, providing structured support for clinician review within the clinical validation workflow prior to any patient-facing use of the SDD.

### Clinical Validation, Delivery, and Follow-Up

Before any patient-facing use, each AI-generated SDD undergoes a qualitative clinical validation performed by the treating physician. This review is aimed at verifying the clinical correctness, completeness, and appropriateness of the generated content with respect to the HDR and the discharge interview. Based on this initial assessment, the SDD is classified as suitable, suitable with minor corrections, or not suitable. Documents classified as not suitable are withheld and are not delivered to the patient. Following the initial review, clinicians complete a structured evaluation inspired by the QUEST (quality, understanding, expression style, safety, and trust) framework [32] to provide a standardized assessment of the AI-generated SDD (Multimedia Appendix 1). This evaluation addresses accuracy, completeness, coherence, clarity, utility, safety, and reliability, together with a structured inventory of any content errors detected, organized into prespecified categories (factual errors, omissions, hallucinations, linguistic issues, contradictions with the source documents, presentation errors, and formatting errors), each with associated branching items capturing subtype and severity, and supplementary items addressing potentially anxiety-inducing formulations, neutrality and absence of stereotypical or discriminatory language, and the perceived potential for the SDD to lead to clinically consequential misinterpretation. This second step is intended to systematically document clinician judgment and to support traceable quality and safety assessment of the generated documents. If deemed suitable, the SDD is delivered to the patient together with the HDR, which remains the only legally valid discharge document. Patients are explicitly informed that the SDD is a supplementary informational tool designed to facilitate understanding of discharge information and postdischarge management and does not replace the HDR. Within 1 month after discharge, patients are invited to complete a structured follow-up questionnaire assessing perceived accessibility, comprehensibility, usefulness, engagement, and overall experience with the SDD (Multimedia Appendix 2). This follow-up is designed to capture patient-reported perceptions of the SDD as a support tool for understanding and managing health information after hospital discharge. In parallel, clinicians receive the CIPR generated by the AI system, which compares the HDR with the transcript of the discharge interview. The report highlights omissions, inconsistencies, and critical information requiring emphasis between written and verbal discharge communication. Clinicians evaluate the perceived utility of this report as a support tool for improving documentation quality and clinician-patient communication through a dedicated structured survey (Multimedia Appendix 3). This assessment also collects information on feasibility, usability, perceived impact on discharge practices, and integration within routine clinical workflows, including perceived time burden. A dedicated linguistic analysis is conducted on the AI-generated SDDs to characterize their accessibility and communicative profile. Readability is assessed using the Gulpease index [38], a validated measure for the Italian language that estimates textual accessibility based on sentence length and word complexity. Gulpease scores are interpreted in relation to patients’ measured HL levels to evaluate alignment between linguistic complexity and user capacity. In addition to readability, a corpus-based linguistic analysis [39] is performed to examine syntactic and discourse-level features of the SDDs. A comparative corpus is constructed, including both AI-generated SDDs and the corresponding original HDRs. Quantitative analyses use computational linguistic tools to extract indicators of lexical density and syntactic complexity [40,41], including measures such as type-token ratio [42] and measure of textual lexical diversity [43]. Qualitative discourse analysis is conducted to examine information structure, cohesion, progression of information, management of given and new information, and adaptation of specialist terminology. These analyses aim to identify linguistic strategies that most effectively support patient comprehension.

### Safety Monitoring of AI-Generated Outputs

A structured framework for the identification, classification, and tracking of AI-related errors is integrated into the clinical validation step (Multimedia Appendix 1). For each AI-generated SDD, the treating physician completes a QUEST-informed structured evaluation that records, in a single instrument, both the perceived quality of the output across QUEST domains and a detailed inventory of any errors detected. Errors are classified into seven prespecified categories, operationalized to capture the principal failure modes relevant to AI-mediated patient-facing communication: (1) factual errors, including incorrect diagnoses, therapies, dosages, laboratory or diagnostic findings, allergy or contraindication information, and follow-up plans; (2) omissions, including missing therapy or postdischarge instructions, missing warnings on side effects or risks, and missing follow-up procedures or appointments; (3) hallucinations, defined as content not supported by the source documents, including unsupported diagnoses, therapies not discussed during the discharge encounter or absent from the HDR, and clinical recommendations not aligned with established guidelines; (4) linguistic issues, including overcomplex terminology, ambiguous formulations, and unsafe oversimplifications that may render instructions vague or imprecise; (5) contradictions, including discrepancies between sections of the SDD and discrepancies with information provided in the HDR or during the discharge encounter; (6) presentation errors, including illogical sequencing or disconnected content; and (7) formatting errors. This taxonomy is intended to address the principal categories of AI-related risk in patient-facing clinical documents, including hallucinations, missing or incorrect medication and follow-up information, unsafe simplifications, and contradictions with the official discharge documentation.

For each error category identified, a branching set of items records the specific subtype and a 5-level severity rating (from 1=not at all severe to 5=extremely severe). Two additional items capture the perceived attribution of inaccuracies or omissions to the source documents, that is, whether the issue may be traced to information provided in the HDR or during the discharge interview, supporting the separation of model-related from input-related errors. Additional items address potentially anxiety-inducing formulations, neutrality and absence of stereotypical or discriminatory language, and the perceived potential for the SDD to lead to clinically consequential misinterpretation. A final item invites clinician reflection on whether the original HDR or discharge interview would have been structured differently in light of the CIPR output, supporting iterative improvement of clinical communication practice.

Documents classified as not suitable during clinical validation are withheld from delivery and are not provided to the patient under any circumstances, regardless of the nature or severity of the detected issue. Documents classified as suitable with minor corrections are revised by the treating physician prior to delivery, and the corrections applied are recorded for subsequent analysis. Aggregated error data (including the frequency and severity distribution of each error category, the proportion of withheld documents, and the corrected components) are reviewed by the research team at prespecified procedural checkpoints corresponding to the completion of approximately 30%, 60%, and 100% of the planned reports within each clinical unit. At each checkpoint, a panel of clinical experts not directly involved in the original validation performs an independent ex-post review of a sample of generated SDDs, providing a second-line assessment of clinical quality and safety. Should patterns suggestive of recurrent or severe AI-related errors emerge (including, but not limited to, repeated hallucinations involving therapeutic content, repeated omissions of follow-up information, or systematic contradictions with the HDR), the research team reassesses the system configuration and documentation workflow before continuing enrollment. No fixed numerical stopping threshold is prespecified, given the exploratory nature of the study and the absence of established benchmarks for this type of system; safety-related decisions and any consequent modifications to the workflow are documented in the study records and reported in the final analysis. Aggregated error frequencies, severity distributions, and procedural decisions taken at each checkpoint are reported alongside the primary outcome results, supporting transparent assessment of the clinical safety profile of the AI system within the study workflow.

### Ethical Considerations

**Figure 2:**
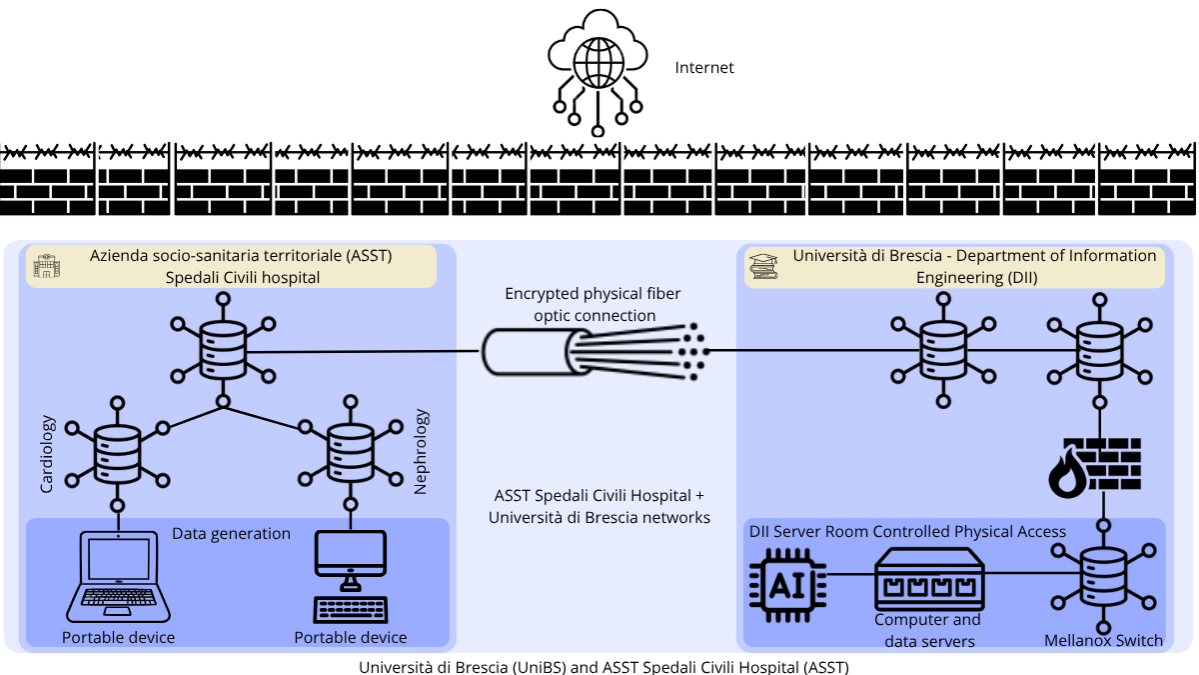
Hospital and university secure network diagram. AI: artificial intelligence; ASST: Azienda Socio-Sanitaria Territoriale; DII: Department of Information Engineering.

The study is conducted in accordance with the principles of the Declaration of Helsinki, Good Clinical Practice guidelines [44], and applicable European and Italian regulatory frameworks governing clinical research, data protection, and the use of AI in health care [45,46]. The study protocol received approval from the Comitato Etico Territoriale Lombardia 6, the reference ethics committee for the participating clinical centers. The 2 institutions jointly contribute to the conduct of the study within their respective roles and responsibilities. A formal favorable resolution authorizing participation in the nonprofit study has been issued by the competent university departmental governance bodies (111-09/04/2025). In compliance with Regulation (EU) 2016/679 (General Data Protection Regulation) [45] and Italian Legislative Decree No 196/2003, as amended by Legislative Decree No 101/2018 [46], a comprehensive Data Protection Impact Assessment (DPIA) has been conducted prior to study initiation. The DPIA was formally adopted by the data controllers following verification by the competent institutional units, and written opinions issued by the respective data protection officers of the University of Brescia and ASST Spedali Civili di Brescia. The DPIA defines and monitors all technical and organizational measures implemented to mitigate risks related to the processing of personal and health data within the study. Roles and responsibilities related to data processing are clearly defined in accordance with General Data Protection Regulation requirements. Data are processed exclusively within secure institutional infrastructures shared between the hospital and the university, with both entities operating under clearly delineated governance arrangements. Access to data is restricted to authorized personnel involved in the study, and all processing activities are logged and auditable. Personal data are pseudonymized using unique alphanumeric identifiers, and identifying information is stored separately from clinical and study data. The AI system processes source documents transiently in memory solely for the purposes of document generation and production of the CIPR. Patient data are not retained within AI models, are not used for model retraining, and are not transferred to external cloud services. Data transmission occurs exclusively through protected internal networks using encrypted connections, and secure backup, incident management, and disaster recovery procedures are in place in accordance with institutional policies and the approved DPIA (Figure 2). Beyond data protection, the study addresses clinical risks specific to the introduction of an AI-generated supplementary document into the postdischarge information pathway. Three principal risks are considered. First, the risk that patients may misinterpret the simplified content of the SDD; second, the risk that patients may rely on the SDD to a greater extent than on the HDR, which remains the only legally valid discharge document; and third, the risk that simplified instructions in the SDD may not fully reflect the clinical detail or qualifications contained in the HDR. To mitigate these risks, several procedural safeguards are implemented within the study workflow. No SDD is delivered to a patient without prior clinical validation by the treating physician, and documents classified as not suitable during validation are withheld from delivery as described in the safety monitoring framework. Linguistic adaptation of the SDD is anchored to the patient’s measured HL level, with the aim of reducing the likelihood of oversimplifications that could render instructions vague or imprecise. At the time of delivery, patients are explicitly informed that the SDD is a supplementary informational tool intended to support understanding of the discharge content, that it does not replace the HDR, and that the HDR remains the reference document for any clinical or administrative purpose. This explanation is provided verbally during the discharge encounter and is also recorded in writing within the SDD itself. Throughout the study, the structured QUEST-informed clinical evaluation (Multimedia Appendix 1) and the safety monitoring framework described earlier support continuous identification, classification, and review of any AI-related issues that could carry clinical implications, including unsafe simplifications and contradictions with the HDR. Patients and clinicians may report concerns regarding the SDD or the discharge process at any point during the study, and such reports are documented and reviewed by the research team. No clinical decision-making is delegated to the AI system at any stage of the workflow, and the treating physician retains full clinical responsibility for the discharge process and for the information communicated to the patient. All participants receive clear and comprehensive information about the study objectives, procedures, potential risks, and data handling practices during an individual information session prior to enrollment. Written informed consent is obtained before any study-related activity is performed. Participation is voluntary, and participants may withdraw consent at any time without any consequences for their ongoing or future clinical care. No compensation will be provided to participants.

### Statistical Analysis

Data analyses are intended to be primarily descriptive and exploratory, consistent with the prospective, observational, single-arm pilot design of the study. Analytic choices are prespecified in alignment with the outcome defined in the study (primary, secondary, and exploratory). Continuous variables are summarized using means and SDs or medians and IQRs, depending on the empirical distribution; categorical variables are summarized using absolute and relative frequencies. Sociodemographic and clinical characteristics, HL scores obtained through the 16-item European Health Literacy Survey Questionnaire and the brief HL screen, and structural characteristics of the source documents are reported descriptively for the overall sample and stratified by clinical unit.

Analyses related to the primary outcome, namely, the clinician-rated quality and safety of the AI-generated SDD, are descriptive in nature and do not involve formal hypothesis testing, consistent with the absence of a comparison group and the pilot scope of the study. The proportion of SDDs classified as suitable, suitable with minor corrections, and not suitable for patient delivery is reported with corresponding 95% CIs, calculated using methods appropriate for binomial proportions. The QUEST-informed assessment items, covering accuracy, completeness, clarity, safety, and the structured taxonomy of content errors, are summarized through medians, IQRs, and response distributions for ordinal items, and absolute and relative frequencies for categorical items. Per-domain results are reported for the overall sample and stratified by clinical unit.

Analyses related to the secondary outcomes are also primarily descriptive, with inferential analyses limited to associations explicitly identified as exploratory. Patient-reported accessibility, comprehensibility, usefulness, and engagement with the SDD, collected approximately 1 month after discharge, are summarized at the item and scale level, with 95% CIs where appropriate. Clinician-rated perceived utility of the CIPR is summarized in an analogous manner. Feasibility and workflow indicators are operationalized a priori as (1) the study completion rate, defined as the proportion of consented participants who complete all study procedures through the 1-month patient follow-up; (2) the proportion of generated SDDs withheld from delivery due to clinical nonsuitability; and (3) clinician-perceived integration burden, measured through the dedicated CIPR questionnaire. Readability of the SDDs, assessed through the Gulpease index, is reported descriptively for the overall corpus and stratified by clinical unit, alongside the readability of the corresponding HDRs.

Analyses related to the exploratory outcomes are intended to support hypothesis generation and to inform the design of future comparative studies; results are interpreted with corresponding caution and are not used to draw conclusions regarding efficacy. Associations between SDD readability (Gulpease index) and patient-reported comprehensibility, and between syntactic-discourse linguistic indicators (including type-token ratio and measure of textual lexical diversity) and patient-reported outcomes, are examined using Pearson or Spearman correlation coefficients, with the choice guided by the empirical distribution and measurement scale of each variable pair. Prespecified subgroup comparisons across clinical units (nephrology vs cardiology, 2 groups) and across HL categories are conducted using independent-sample 2-tailed *t* tests or Mann-Whitney *U* tests and 1-way analysis of variance or Kruskal-Wallis tests, respectively, with the choice between parametric and nonparametric forms guided by distributional and variance assumptions; all tests are 2-tailed [47]. Where data quality and distributional characteristics permit, exploratory multivariable regression models may be considered to describe joint associations; the form of the model (linear, logistic, and ordinal) is selected on the basis of the outcome distribution. Such models are intended to be descriptive and hypothesis-generating, not to support causal or predictive inference.

All inferential analyses within the exploratory layer are 2-sided, with a nominal significance threshold of *P*<.05 reported alongside point estimates and 95% CIs. No formal adjustment for multiple comparisons is prespecified, in line with the descriptive and hypothesis-generating intent of the analyses; results from analyses involving multiple comparisons are interpreted with explicit caution and are not presented as confirmatory. No interim analyses are planned. Analyses are conducted on pseudonymized data using established statistical software. Missing data are summarized descriptively per variable; no imputation is prespecified, and analyses involving variables with substantial missingness are interpreted with corresponding caution.

## Results

The study protocol received approval from the reference ethics committee and institutional review board (Comitato Etico Territoriale Lombardia 6) for the participating clinical centers, during the committee meeting held on February 24, 2026 (authorization: AIM-HEALTH NP 6761-NP 6762). A DPIA has been completed and formally adopted following institutional verification, and written opinions issued by the data protection officers of both the hospital and the university (Prot 127620/07.05.25). Formal institutional authorizations have been obtained from the participating units and university governance bodies. The project is supported by a competitive research grant awarded by Fondazione Cariplo (Bando Giovani Ricercatori 2025, grant protocol 2025-616/SH), covering the years 2026-2028. Data collection commenced on May 29, 2026.

## Discussion

Hospital discharge communication represents a critical transition point in the care pathway, particularly for patients with chronic conditions who must manage complex therapeutic regimens after hospitalization. Variability in HL and the linguistic complexity of HDRs can hinder understanding and contribute to fragmented continuity of care. The AIM-HEALTH (Artificial Intelligence–Mediated Discharge Document for Accessible Healthcare) study aims to address this gap by evaluating a structured, clinician-validated approach to generating SDDs using AI within routine hospital workflows. A key strength of the study lies in its dual-output design. By pairing HL-adapted SDDs with a CIPR, the protocol explicitly separates patient-facing support from tools intended to assist clinicians in improving documentation quality and discharge communication. This separation preserves the legal and clinical primacy of the HDR while enabling targeted informational support and systematic identification of discrepancies between written and verbal communication. The integration of mandatory clinician validation and standardized quality assessment further reinforces clinical governance and safety. The study also contributes to the emerging literature on the responsible implementation of AI in health care settings. The exclusive use of on-premise infrastructure, the absence of model retraining on patient data, and the integration of data protection safeguards defined through a formal DPIA reflect a governance-oriented approach that aligns with European regulatory requirements. In this respect, the protocol offers a replicable framework for institutions seeking to explore AI-mediated health communication while maintaining accountability, transparency, and compliance with ethical and legal standards. Several limitations should be acknowledged. The study is conducted in a single tertiary hospital and focuses on 2 clinical units, which may limit generalizability to other settings or specialties. In addition, the observational design does not allow causal inference regarding the impact of the SDD on clinical outcomes. However, as a protocol study, AIM-HEALTH is intended to generate feasibility data, inform refinement of AI-supported discharge processes, and support the design of future comparative or multicenter investigations. If successfully implemented, the AIM-HEALTH protocol may inform broader organizational strategies aimed at improving discharge communication, supporting patient understanding, and strengthening continuity of care through ethically governed, clinician-centered AI tools.

## Data Availability

The datasets generated or analyzed during this study are not publicly available due to ethical, legal, and data protection constraints related to the processing of sensitive health data but are available from the corresponding author on reasonable request.

## Appendix

Multimedia Appendix 1 Quality, understanding, expression style, safety, and trust–informed structured evaluation tool.

Multimedia Appendix 2 Patient survey for the evaluation of the supplementary discharge document.

Multimedia Appendix 3 Clinical survey.

Multimedia Appendix 4 Peer review report by Bando Giovani Ricercatori 2025 (Young Researchers – 2025) Review Committee, Fondazione Cariplo (Italy).

## Abbreviations

AI: artificial intelligence
AIM-HEALTH: Artificial Intelligence–Mediated Discharge Document for Accessible Healthcare
ASST: Azienda Socio-Sanitaria Territoriale
CIPR: clinical informational performance report
DPIA: Data Protection Impact Assessment
HDR: hospital discharge report
HL: health literacy
LLM: large language model
QUEST: quality, understanding, expression style, safety, and trust
SDD: supplementary discharge document

## References

[1] Weetman K, Spencer R, Dale J, Scott E, Schnurr S (2021). What makes a “successful” or “unsuccessful” discharge letter? Hospital clinician and General Practitioner assessments of the quality of discharge letters. BMC Health Serv Res.

[2] Piepenhagen G, Röhrig B, Eirund W, Roth-Sackenheim C, Steffens M (2021). The importance of high quality discharge letters: an empirical investigation. Gesundheitswesen.

[3] McCarthy DM, Waite KR, Curtis LM, Engel KG, Baker DW, Wolf MS (2012). What did the doctor say? Health literacy and recall of medical instructions. Med Care.

[4] Burns ST, Amobi N, Chen JV, O'Brien M, Haber LA (2022). Readability of patient discharge instructions. J Gen Intern Med.

[5] Schwarz CM, Hoffmann M, Schwarz P, Kamolz L, Brunner G, Sendlhofer G (2019). A systematic literature review and narrative synthesis on the risks of medical discharge letters for patients' safety. BMC Health Serv Res.

[6] Weetman K, Wong G, Scott L, Kearney F (2017). Adherence to hospital discharge medication: a qualitative study of patients' and healthcare professionals' experiences. BMJ Open.

[7] Sørensen K, Pelikan JM, Röthlin F, Ganahl K, Slonska Z, Doyle G, Fullam J, Kondilis B, Agrafiotis D, Uiters E, Falcon M, Mensing M, Tchamov K, Broucke SVD, Brand H (2015). Health literacy in Europe: comparative results of the European health literacy survey (HLS-EU). Eur J Public Health.

[8] Organisation for Economic Co-operation and Development (2019). OECD Skills Outlook 2019: Thriving in a Digital World.

[9] Petruzzelli D, Vignetti M, Trasarti S, Sportoletti P, Torre SD, Cairoli R, Leone FPC, Pompilio G, Gullì M, Hajdukova EB, Integlia D (2024). Exploring the administrative burden faced by hematologists: a comprehensive study in Italy. Glob Reg Health Technol Assess.

[10] Pelizzari N, Covolo L, Ceretti E, Fiammenghi C, Gelatti U (2025). Defining, assessing, and implementing organizational health literacy: barriers, facilitators, and tools—a systematic review. BMC Health Serv Res.

[11] Carroll AR, Johnson JA, Stassun JC, Greevy RA, Mixon AS, Williams DJ (2024). Health literacy-informed communication to reduce discharge medication errors in hospitalized children: a randomized clinical trial. JAMA Netw Open.

[12] Watson DE, Marashi-Pour S, Tran B, Witchard A (2022). Patient-reported experiences and outcomes following hospital care are associated with risk of readmission among adults with chronic health conditions. PLoS One.

[13] Brunner-La Rocca HP, Peden CJ, Soong J, Holman PA, Bogdanovskaya M, Barclay L (2020). Reasons for readmission after hospital discharge in patients with chronic diseases—information from an international dataset. PLoS One.

[14] Sheikhalishahi S, Miotto R, Dudley JT, Lavelli A, Rinaldi F, Osmani V (2019). Natural language processing of clinical notes on chronic diseases: systematic review. JMIR Med Inform.

[15] Amin KS, Davis MA, Doshi R, Haims AH, Khosla P, Forman HP (2023). Accuracy of ChatGPT, Google Bard, and Microsoft Bing for simplifying radiology reports. Radiology.

[16] Dubinski D, Won SY, Trnovec S, Behmanesh B, Baumgarten P, Dinc N, Konczalla J, Chan A, Bernstock JD, Freiman TM (2024). Leveraging artificial intelligence in neurosurgery—unveiling ChatGPT for neurosurgical discharge summaries and operative reports. Acta Neurochir.

[17] Barak-Corren Y, Wolf R, Rozenblum R, Creedon JK, Lipsett SC, Lyons TW, Michelson KA, Miller KA, Shapiro D, Reis BY, Fine AM (2024). Harnessing the power of generative AI for clinical summaries: perspectives from emergency physicians. Ann Emerg Med.

[18] Ando K, Okumura T, Komachi M, Horiguchi H, Matsumoto Y (2022). Is artificial intelligence capable of generating hospital discharge summaries from inpatient records?. PLoS Digit Health.

[19] Ali SR, Dobbs TD, Hutchings HA, Whitaker IS (2023). Using ChatGPT to write patient clinic letters. Lancet Digit Health.

[20] Ayre J, Mac O, McCaffery K, McKay BR, Liu M, Shi Y, Rezwan A, Dunn AG (2024). New frontiers in health literacy: using ChatGPT to simplify health information for people in the community. J Gen Intern Med.

[21] Clough RAJ, Sparkes WA, Clough OT, Sykes JT, Steventon AT, King K (2023). Transforming healthcare documentation: harnessing the potential of AI to generate discharge summaries. BJGP Open.

[22] Patel SB, Lam K (2023). ChatGPT: the future of discharge summaries?. Lancet Digit Health.

[23] Jeblick K, Schachtner B, Dexl J, Mittermeier A, Stüber AT, Topalis J, Weber T, Wesp P, Sabel BO, Ricke J, Ingrisch M (2024). ChatGPT makes medicine easy to swallow: an exploratory case study on simplified radiology reports. Eur Radiol.

[24] Van Veen D, Van Uden C, Blankemeier L, Delbrouck J, Aali A, Bluethgen C, Pareek A, Polacin M, Reis EP, Seehofnerová A, Rohatgi N, Hosamani P, Collins W, Ahuja N, Langlotz CP, Hom J, Gatidis S, Pauly J, Chaudhari AS (2024). Adapted large language models can outperform medical experts in clinical text summarization. Nat Med.

[25] Nayak A, Alkaitis MS, Nayak K, Nikolov M, Weinfurt KP, Schulman K (2023). Comparison of history of present illness summaries generated by a chatbot and senior internal medicine residents. JAMA Intern Med.

[26] Huang T, Safranek C, Socrates V, Chartash D, Wright D, Dilip M, Sangal RB, Taylor RA (2024). Patient-representing population's perceptions of GPT-Generated versus standard emergency department discharge instructions: randomized blind survey assessment. J Med Internet Res.

[27] Gimeno A, Krause K, D'Souza S, Walsh C (2024). Completeness and readability of GPT-4-generated multilingual discharge instructions in the pediatric emergency department. JAMIA Open.

[28] Sánchez-Rosenberg G, Magnéli M, Barle N, Kontakis MG, Müller AM, Wittauer M, Gordon M, Brodén C (2024). ChatGPT-4 generates orthopedic discharge documents faster than humans maintaining comparable quality: a pilot study of 6 cases. Acta Orthop.

[29] Schwieger A, Angst K, de Bardeci M, Burrer A, Cathomas F, Ferrea S, Grätz F, Knorr M, Kronenberg G, Spiller T, Troi D, Seifritz E, Weber S, Olbrich S (2024). Large language models can support generation of standardized discharge summaries—a retrospective study utilizing ChatGPT-4 and electronic health records. Int J Med Inform.

[30] Kim J, Dunn AG, Ayre J, Khadra S, Tracy M, Koria L, Lo S, Naganathan V, Kim J, Dunn AG, Ayre J (2024). The quality and safety of using generative AI to produce patient-centred discharge instructions. NPJ Digit Med.

[31] Schmitz B (2023). Improving accessibility of scientific research by artificial intelligence—an example for lay abstract generation. Digit Health.

[32] Tam TYC, Sivarajkumar S, Kapoor S, Stolyar AV, Polanska K, McCarthy KR, Osterhoudt H, Wu X, Visweswaran S, Fu S, Mathur P, Cacciamani GE, Sun C, Peng Y, Wang Y (2024). A framework for human evaluation of large language models in healthcare derived from literature review. npj Digit. Med.

[33] Hertzog MA (2008). Considerations in determining sample size for pilot studies. Res Nurs Health.

[34] Lancaster GA, Dodd S, Williamson PR (2004). Design and analysis of pilot studies: recommendations for good practice. J Eval Clin Pract.

[35] Eldridge SM, Lancaster GA, Campbell MJ, Thabane L, Hopewell S, Coleman CL, Bond CM (2016). Defining feasibility and pilot studies in preparation for randomised controlled trials: development of a conceptual framework. PLoS One.

[36] Lorini C, Santomauro F, Grazzini M, Mantwill S, Vettori V, Lastrucci V, Bechini A, Boccalini S, Bussotti A, Bonaccorsi G (2017). Health literacy in Italy: a cross-sectional study protocol to assess the health literacy level in a population-based sample, and to validate health literacy measures in the Italian language. BMJ Open.

[37] Wang S, Yang CHH, Wu J, Zhang C (2024). Can whisper perform speech-based in-context learning?.

[38] Lucisano P, Piemontese ME (1988). GULPEASE: una formula per la predizione della difficoltà dei testi in lingua italiana [GULPEASE: a formula for predicting the difficulty of Italian-language texts]. Scuola Città.

[39] Baker P, Gabrielatos C, KhosraviNik M, Krzyżanowski M, McEnery T, Wodak R (2008). A useful methodological synergy? Combining critical discourse analysis and corpus linguistics to examine discourses of refugees and asylum seekers in the UK press. Discourse Soc.

[40] To V, Fan S, Thomas D (2013). Lexical density and readability: a case study of English textbooks. Internet J Language Cult Soc.

[41] Tweedie FJ, Baayen RH (1998). How variable may a constant be? Measures of lexical richness in perspective. Comput Humanit.

[42] Kettunen K (2014). Can type-token ratio be used to show morphological complexity of languages?. J Quant Linguist.

[43] McCarthy PM, Jarvis S (2010). MTLD, vocd-D, and HD-D: a validation study of sophisticated approaches to lexical diversity assessment. Behav Res Methods.

[44] World Medical Association (2013). World Medical Association Declaration of Helsinki: ethical principles for medical research involving human subjects. JAMA.

[45] European Parliament and Council of the European Union (2016). Regulation (EU) 2016/679 of the European Parliament and of the Council of 27 April 2016 on the protection of natural persons with regard to the processing of personal data and on the free movement of such data, and repealing Directive 95/46/EC (General Data Protection Regulation). Official Journal of the European Union.

[46] Italian Republic (2026). Legislative decree No. 196 of 30 June 2003, as amended by legislative decree No. 101 of 10 August 2018. Personal Data Protection Code. Gazzetta Ufficiale della Repubblica Italiana.

[47] Field AP (2018). Discovering Statistics Using IBM SPSS Statistics. 5th Edition.

