# Supplementary material for: Artificial Intelligence–Mediated Discharge Document for Accessible Health Care (AIM-HEALTH): Protocol for a Prospective, Observational, Noninterventional Study
Source: JMIR Res Protoc. 2026 Jul 3;15:e95782. doi: 10.2196/95782 (PMC13379690; doi:10.2196/95782)
Supplement: Multimedia Appendix 1 [file resprot_v15i1e95782_app1.docx]

**QUEST-informed Structured Evaluation Tool**

**Purpose and Structure**

This questionnaire is administered to the clinician responsible for the AI-generated discharge content within the AIM-HEALTH study. Its purpose is to support a structured, traceable evaluation of the AI-generated supplementary discharge document (SDD) prior to any patient-facing use, and to document the clinician judgement underpinning the suitability classification of each generated output. The instrument is used as a single record per generated SDD and combines closed-format ratings with branching items capturing the type and severity of any errors identified.

Each item is completed by selecting the most appropriate option. Likert-scale items use a five-point agreement scale; non-agreement items use the most appropriate single response. Branching items appear conditionally on prior responses, allowing the clinician to characterise the type and severity of detected errors in detail.

This document provides a printable version of the questionnaire, which is administered electronically through an automated platform supporting all data collection procedures within the study.

The questionnaire is informed by the QUEST framework for the human evaluation of large language models in healthcare (Tam et al., 2024).

**Administrative Information**

**Report generated at:**

- Nephrology Unit
- Cardiology Unit

**Report identification code:** ____________________ *(e.g., N01, C05)*

**Operator (clinician) identifier:** ____________________

**Items**

**Q1. The AI-generated report is accurate and clinically and scientifically correct.**

- Strongly agree
- Agree
- Neither agree nor disagree
- Disagree
- Strongly disagree

**Q2. Does the AI-generated report contain factual errors or relevant omissions?**

- Yes
- No
- Other (please specify)

*If “Yes” is selected, the branching logic below applies to identify the type(s) of error.*

**Q2.1 Which type of scenario was observed? (multiple selection allowed)**

- Factual error (incorrect clinical or therapeutic information)
- Omission of essential information
- Fabricated content not supported by the source documents (hallucination)
- Inappropriate use of language / ambiguous language
- Contradiction among the information provided
- Information presentation error (confused order, disconnected information)
- Formatting or layout error (e.g., truncated text, incorrect numbering)

*Conditional follow-up items appear based on the scenarios selected.*

**Q2.2 If a factual error was identified — which type of factual error was observed? (multiple selection allowed)**

- Incorrect diagnosis
- Incorrect therapy
- Incorrect dosage
- Error in laboratory data or diagnostic test results
- Error in the indication of allergies or contraindications
- Error in the follow-up plan or post-discharge recommendations
- Other (please specify)

**Q2.3 How severe is this factual error?**

- Not at all severe
- Slightly severe
- Moderately severe
- Very severe
- Extremely severe

**Q2.4 If an omission was identified — which type of information was omitted? (multiple selection allowed)**

- Relevant clinical data
- Prescribed or recommended therapy
- Instructions for post-discharge management
- Warnings on potential side effects or risks
- Information on when and how to contact the physician
- Missing follow-up procedures or appointments
- Other (please specify)

**Q2.5 How severe is this omission?**

- Not at all severe
- Slightly severe
- Moderately severe
- Very severe
- Extremely severe

**Q2.6 If a hallucination was identified — what type of information was fabricated by the AI system? (multiple selection allowed)**

- Diagnosis not supported by the clinical data
- Therapy never discussed with the patient or absent from the HDR
- Diagnostic tests not performed but reported in the document
- Incorrect or non-existent personal or contact details
- Recommendations not aligned with established clinical guidelines
- Other (please specify)

**Q2.7 How severe is this hallucination?**

- Not at all severe
- Slightly severe
- Moderately severe
- Very severe
- Extremely severe

**Q2.8 If a linguistic issue was identified — which type of linguistic issue was observed? (multiple selection allowed)**

- Medical terms too complex for the patient
- Ambiguous or unclear sentences
- Grammatical or stylistic errors
- Ambiguity regarding who should perform an action (e.g., patient vs. physician)
- Excessive simplification of language rendering instructions vague
- Inappropriate use of unexplained abbreviations or acronyms
- Other (please specify)

**Q2.9 Do you consider that this issue could compromise comprehension of the report?**

- Yes
- No

**Q2.10 If a contradiction was identified — which type of contradiction was observed? (multiple selection allowed)**

- Clinical data that contradict each other (e.g., inconsistent diagnosis and therapy)
- Differences between therapeutic recommendations
- Discordant indications across different sections of the report
- Information differing from that provided verbally or in the HDR
- Other (please specify)

**Q2.11 Do you consider that this issue could compromise comprehension of the report?**

- Yes
- No

**Q2.12 If a presentation issue was identified — which type of presentation issue was observed? (multiple selection allowed)**

- Confused or non-logical order of information
- Unnecessary repetition of information across different sections
- Information that is disconnected from related content
- Missing transitions making the logic of the report difficult to follow
- Other (please specify)

**Q2.13 Do you consider that this issue could compromise comprehension of the report?**

- Yes
- No

**Q2.14 If a formatting/layout error was identified — which type of formatting error was observed?**

- Truncated text or missing parts of the document
- Numbering errors or incorrect headings
- Incorrect indentation or spacing impairing readability
- Errors in references to figures or tables (e.g., a table cited but absent)
- Other (please specify)

**Q2.15 Do you consider that this issue could compromise comprehension of the report?**

- Yes
- No

**Q3. To what extent do you consider that any inaccuracies or omissions in the report are attributable to the information provided in the hospital discharge report (HDR)?**

- Not at all
- Slightly
- Moderately
- Very much
- Completely

**Q4. To what extent do you consider that any inaccuracies or omissions in the report are attributable to the information provided during the discharge interview?**

- Not at all
- Slightly
- Moderately
- Very much
- Completely

*For the following items, branching logic applies based on the response.*

**Q5. Does the report contain irrelevant information?**

- Yes
- No

*If “Yes”: open-ended item — Which irrelevant information was identified?*

**Q6. Is the report formulated in a clear and comprehensible way?**

- Yes
- No

*If “No”: open-ended item — Which sections do you consider to have been unclear or difficult to understand?*

**Q7. Does the report contain specific formulations that could generate anxiety or excessive concern?**

- Yes
- No

*If “Yes”: open-ended item — Which formulation do you consider could potentially generate anxiety or excessive concern?*

**Q8. Do you consider that the report is written in a neutral and impartial way, avoiding stereotypes or biases that could influence the patient’s perception?**

*For example, it avoids expressions that could imply judgements regarding the patient’s responsibility (e.g., “the patient did not follow the therapy correctly” instead of “the therapy was not followed as indicated”), or that attribute negative characteristics to specific categories of persons.*

- Yes
- No

*If “No”: open-ended item — Which formulation do you consider could have influenced the patient’s perception?*

**Q9. Do you consider that the report contains expressions or formulations that could result in discrimination towards the patient or specific groups of persons?**

*For example, it contains unnecessary references to age, gender, nationality, or other aspects that are not clinically relevant (e.g., “elderly patient with poor adherence to therapy” instead of “the patient presents difficulty in following the therapy”).*

- Yes
- No

*If “Yes”: open-ended item — Which formulation do you consider could result in discrimination?*

**Q10. Do you consider that the report could lead to misinterpretations with possible negative consequences for the patient, such as inappropriate clinical decisions, confusion regarding therapeutic instructions, or non-adherence to treatment?**

- Strongly disagree
- Disagree
- Neither agree nor disagree
- Agree
- Strongly agree

**Q11. In light of the report on clinical informational performance, would you, in retrospect, have structured the HDR or the discharge interview differently?**

- Yes, both
- Yes, the HDR
- Yes, the discharge interview
- No

**Reference**

Tam TYC, Sivarajkumar S, Kapoor S, Stolyar AV, Polanska K, McCarthy KR, Osterhoudt H, Wu X, Visweswaran S, Fu S, Mathur P, Cacciamani GE, Sun C, Peng Y, Wang Y. A framework for human evaluation of large language models in healthcare derived from literature review. *NPJ Digital Medicine.* 2024;7(1):258. doi:10.1038/s41746-024-01258-7
