# Supplementary material for: Artificial Intelligence–Mediated Discharge Document for Accessible Health Care (AIM-HEALTH): Protocol for a Prospective, Observational, Noninterventional Study
Source: JMIR Res Protoc. 2026 Jul 3;15:e95782. doi: 10.2196/95782 (PMC13379690; doi:10.2196/95782)
Supplement: Multimedia Appendix 2 [file resprot_v15i1e95782_app2.docx]

**Patient Survey for the Evaluation of the SDD**

*(English translation)*

Dear Patient,

We invite you to complete this survey to help us evaluate your experience with the AIM-HEALTH document in supporting you in managing your health after discharge. Your contribution is valuable in improving the quality of information provided to patients.

| **How to complete the questionnaire:**   - Answer the questions based on your experience with the AIM-HEALTH document. - Select the answer that best represents your opinion. - There are no right or wrong answers; we want to know about your personal experience. |
| --- |

If you have difficulty understanding a question, you may ask the healthcare staff for clarification.

**Thank you for your time!**

**1. How easy was it to understand the information contained in the AIM-HEALTH document?**

- Very easy
- Easy
- Fairly easy
- Difficult
- Very difficult

**2. Compared to the traditional discharge letter, do you think the AIM-HEALTH document is easier to understand?**

- Yes
- No

**3. Was the AIM-HEALTH document helpful in understanding the recommendations regarding your health after discharge?**

- Yes
- No

**4. How would you rate the language used in the AIM-HEALTH document?**

- Very clear
- Clear
- Fairly clear
- Complex
- Very complex

**5. How often did you find terms or phrases that were difficult to understand in the AIM-HEALTH document?**

- Very often (more than 7 times)
- Often (5–6 times)
- Sometimes (3–4 times)
- Rarely (1–2 times)
- Never (0 times)

**6. How clear did you find the structure and organisation of the document?**

- Very clear
- Clear
- Fairly clear
- Confusing
- Very confusing

**7. Which tool did you find most useful and clear for managing your health after discharge: the hospital discharge letter, the AIM-HEALTH document, or the discharge consultation?**

- None were useful or clear
- All tools were equally useful
- The traditional discharge letter
- AIM-HEALTH
- The discharge consultation

**8. How many times did you need to consult the AIM-HEALTH document to recall or better understand information provided in the discharge letter or during the discharge consultation?**

- Very often (more than 7 times)
- Often (5–6 times)
- Sometimes (3–4 times)
- Rarely (1–2 times)
- Never (0 times)

**9. How often did you need to ask the healthcare staff for further clarification after reading the AIM-HEALTH document?**

- Always
- Often
- Sometimes
- Rarely
- Never

**10. How much did the AIM-HEALTH document help you feel more independent and confident in managing your health after discharge?**

- A great deal
- A lot
- Neither much nor little
- A little
- Not at all
