# Supplementary material for: Artificial Intelligence–Mediated Discharge Document for Accessible Health Care (AIM-HEALTH): Protocol for a Prospective, Observational, Noninterventional Study
Source: JMIR Res Protoc. 2026 Jul 3;15:e95782. doi: 10.2196/95782 (PMC13379690; doi:10.2196/95782)
Supplement: Multimedia Appendix 3 [file resprot_v15i1e95782_app3.docx]

**Clinical Survey**

Dear colleague,

The following questions are designed to gather your professional experience with the AIM-HEALTH document. Please feel free to respond with as much or as little detail as you wish: there are no right or wrong answers, and all perspectives are equally valuable.

**Thank you for your time!**

**1. What aspects of the AI-generated report do you find most useful for your clinical practice?**

*(open question)*

**2. Can you provide examples of cases in which the report facilitated post-discharge follow-up?**

*(open question)*

**3. Are the interface and formatting of the AI-generated report functional for your review? What would you improve?**

**4. Have you noticed changes in the quality of patient follow-up thanks to the AIM-HEALTH document?**

*(examples)*

**5. In the event of errors in the AI report, do you believe they are more frequently attributable to the discharge letter (LDO) or to the consultation? Why?**

*(comparative discussion)*

**6. What essential information do you feel is often missing from the discharge letter (LDO) and cannot be retrieved by the AI?**

*(open question)*

**7. What suggestions would you propose to improve the AI system in generating reports?**

*(open question)*

**8. Do you have any suggestions for improving the integration between the AIM-HEALTH document and the traditional discharge letter?**

**9. Do you think an interface with visual summaries or highlighted sections would be useful (e.g. a summary of post-discharge recommendations)?**

**10. Does the balance between patient benefits and the workload required of the clinician to generate the simplified report justify its implementation in clinical practice?**

**11. Do you think the report could be useful in improving communication with the patient?**
