# Supplementary material for: Artificial Intelligence–Mediated Discharge Document for Accessible Health Care (AIM-HEALTH): Protocol for a Prospective, Observational, Noninterventional Study
Source: JMIR Res Protoc. 2026 Jul 3;15:e95782. doi: 10.2196/95782 (PMC13379690; doi:10.2196/95782)
Supplement: Multimedia Appendix 4 [file resprot_v15i1e95782_app4.pdf]

Notes for the Applicant Institution

In response to the *Young Researchers – 2025* call, a total of 662 Letters of Intent (LOIs) were received.

For the evaluation of each LOI, two international experts were consulted and asked to provide an assessment based on the following three criteria:

1. the coherence of the project with the objectives of the call and the relevant research areas;
2. the clarity of presentation and overall quality of the proposal;
3. the suitability of the young researcher to conduct the research independently and their potential for career development.

Each evaluator also provided an overall assessment of the proposal.

Based on these evaluations, only 238 LOIs were admitted to the second stage of evaluation, and your project is among those selected.

Below are the instructions for submitting the application for the second stage.

By accessing the Reserved Area, you will find your project marked as "in progress." You will need to complete several mandatory fields and upload the required documentation.

The mandatory documents to be submitted for the merit evaluation, in the specified format, are as follows:

1. **Cover letter** from the lead institution (also on behalf of partners, in the case of a consortium). The cover letter can be downloaded at:

   https://www.fondazionecariplo.it/it/bandi/Bandi.html

   Please use the "Cover Letter Template," which includes the requested funding amount.

2. **Full proposal (PDF)** – IMPORTANT: follow the instructions provided in the Word template before generating the PDF. The Word version of the full proposal is available in the Reserved Area of the portal.
3. **Budget plan (Excel)** – The budget must be downloaded and uploaded in the section *Project > Actions and Budget Plan*.

In order to submit the project to the second stage of evaluation, all participating organisations (lead institution and partners) must ensure that their approved and signed financial statements are fully updated in the *Organisation Profile* section. It is therefore recommended to verify this with the relevant grant offices and/or administrative departments.

For further information, please consult the FAQ section on the Foundation's website.

The funding application must be submitted no later than 17:00 on the following dates:

- **23 July** for the *Physical Sciences and Engineering* area
- **24 July** for the *Social Sciences and Humanities* area
- **25 July** for the *Life Sciences* area

For any further clarification, please contact:

Physical Sciences and Engineering

Rita Bacchella: ritabacchella@fondazionecariplo.it

Chiara Casella: chiaracasella@fondazionecariplo.it

Social Sciences and Humanities

Valentina Amorese: valentinaamorese@fondazionecariplo.it

Francesca Pierini: francescapierini@fondazionecariplo.it

Life Sciences

Lucia Brambilla: luciabrambilla@fondazionecariplo.it

Alice Finotello: alicefinotello@fondazionecariplo.it

REFEREE 1

RELEVANCE	
The project is interdisciplinary and highly relevant to several ERC domains (e.g., SH4_9, SH7_4, LS7_14, and PE6_11, among others). The project will also help advance the PI's research career by allowing them to extend their expertise in applied linguistics and corpus linguistics into a new domain.	

RESEARCH PROJECT	
The project clearly outlines it research questions, aims, methods, and expected results. The project is interdisciplinary and will have useful implications.	

PI QUALIFICATIONS	
The PI has relevant training in applied linguistics and corpus linguistics and experience in working in the medical domain, positioning them well for the interdisciplinary project. The PI also has a very good publication record.	

OVERALL JUDGMENT AND SUGGESTIONS	
This is a very strong project that will have very useful practical implications. It is unclear to me whether the PI intends to employ fine-tuning in developing the system. If not, I would strongly recommend that they consider doing so in Phase 2.	

REFEREE 2

RELEVANCE

The project addresses a key healthcare challenge: improving patient comprehension of discharge documents through AI-driven solutions. It enhances health communication by adapting medical language for diverse HL levels and evaluating AI tools for accuracy, feasibility, and ethical application in hospital workflows. Findings will be shared in scholarly journals and conferences. This project advances the PI transition to NLP for health outcomes. Collaboration with a highly multidisciplinary team will shape his future as a researcher, combining applied linguistics with the healthcare context for practical applications.

RESEARCH PROJECT

The AIM-Health project proposal demonstrates high clarity and strong quality. The problem is well-defined and significant, the proposed solution is innovative and technologically sound, the methodology is rigorous and well-planned, the interdisciplinary team is a strength, and the potential for both academic and translational impact is clearly articulated. This proposal appears to be a well-conceived and promising research endeavor with the potential to significantly improve hospital discharge communication and patient outcomes, particularly for those with low health literacy.

PI QUALIFICATIONS

The PI has a multidisciplinary knowledge skills and experience.

OVERALL JUDGMENT AND SUGGESTIONS

The AIM-Health project presents several significant strengths. Firstly, its clear and compelling relevance to a critical healthcare problem – the inaccessibility of hospital discharge reports for patients with low health literacy – is a major strength. The proposal effectively uses statistics to highlight the prevalence of this issue and its potential negative consequences. Secondly, the project offers an innovative and timely solution by leveraging the capabilities of AI and large language models to generate patient-centered discharge documents. This approach has the potential to significantly improve patient understanding and adherence to post-discharge care. Thirdly, the well-defined research questions directly address the key aspects of feasibility, clinician assessment, patient perception, and the potential for AI to enhance clinical communication. Fourthly, the methodological rigor of the proposed prospective observational study, including physician validation, the use of validated questionnaires, and the planned analysis of readability metrics, strengthens the scientific validity of the project. The inclusion of a control group (patients receiving standard HDRs) for comparison, though implied, would further enhance the study's design. Furthermore, the explicitly interdisciplinary nature of the research team, bringing together expertise from medicine, AI, linguistics, public health, and legal medicine, ensures a comprehensive and well-rounded approach to the complex challenges involved. Finally, the project demonstrates a clear understanding of translational outcomes and outlines a plan for broader application and policy implications, highlighting the potential for real-world impact beyond academic contributions. The consideration of ethical and data protection regulations (GDPR) is also a crucial strength.

I have some minor suggestions for Phase II: the reliance on physician validation as the primary measure of clinical accuracy might be subjective and could benefit from more specific criteria or a more structured assessment process beyond the suitability ratings. The detailed qualitative clinical assessment using the QUEST-based questionnaire comes later in the study, potentially delaying the identification of critical flaws in the AI-generated reports. The assessment of patient-perceived accessibility and comprehension relies on self-reported questionnaires, which may be subject to biases. While correlating these with objective readability metrics is a good step, incorporating additional measures of actual comprehension could strengthen this aspect. The practical challenges of integrating such an AI tool into existing hospital workflows and the potential impact on clinician workload beyond the stated streamlining effect require further consideration.

Dear Researcher,

We are transmitting the evaluations from the independent international reviewers regarding the project submitted to the "Young Researchers" call for proposals – 2025 edition.

As indicated in the call for proposals, each project was evaluated by a panel of three independent international experts, asked to express a judgment on the following criteria:

1. Scientific quality of the project
2. Adequacy of the profile of the scientific coordinator and the team
3. Impact and dissemination
4. Financial plan and duration

For each criterion, comments were provided and scores were assigned on a scale from 0 to 4 (0 = inadequate; 1 = fair; 2 = good; 3 = excellent; 4 = outstanding). The consistency of the financial plan was assessed by the Foundation's Offices.

The call received a high number of applications characterized by a very high scientific level, making the selection particularly competitive. As envisaged, the overall budget was distributed proportionally among the three disciplinary areas based on the number of applications received, in order to standardize the success rate.

The funding thresholds and the distribution of contributions are therefore as follows:

- **20 projects** fall within the **Life Sciences** area – funding threshold: **89.4 points**
- **12 projects** fall within the **Physical Sciences and Engineering** area – funding threshold: **93.3 points**
- **13 projects** fall within the **Social Sciences and Humanities** area – funding threshold: **93.3 points**

The Offices remain available for any clarifications at the following address: ricercagiovani@fondazionecariplo.it

Information on the next edition of the call will be communicated through the Foundation's website.

Kind regards

DATI GENERALI		
**Codice**	2025-0616	
**Organizzazione**	Università degli Studi di Brescia	
**Sede legale**	BRESCIA (BRESCIA)	
**Legale rappresentante**	Francesco Castelli	
**Località di intervento**	BRESCIA	
**Stato**	Ammissibile	
**Titolo**	AI-mediated discharge document for accessible healthcare (AIM-Health)	
**Responsabile di progetto**	Nicola Pelizzari	
**Descrizione**		
Le lettere di dimissione ospedaliera (LDO) sono essenziali per la gestione post-ricovero, poiché forniscono istruzioni sul follow-up, corretta assunzione della terapia e sul monitoraggio dei sintomi. Tuttavia, la loro efficacia è spesso compromessa da un linguaggio complesso, terminologia specialistica e disomogeneità strutturali, ostacolando la comprensione, soprattutto nei pazienti con livelli di health literacy (HL) inadeguati. Il progetto sviluppa e valida un documento di supporto all'LDO generato tramite un sistema di intelligenza artificiale (IA) appositamente istruito, che integri dati strutturati (LDO, HL) e non strutturati (colloqui clinici) per migliorarne l'accessibilità. Il processo prevede validazione clinica, feedback dei pazienti e analisi linguistica. L'impatto atteso è clinico, migliorando qualità e continuità assistenziale, e scientifico, avanzando l'applicazione dell'IA in sanità. Il progetto promuove soluzioni IA etiche e inclusive in linea con le direttive UE.		

REFEREE 1

SCIENTIFIC QUALITY OF THE PROJECT	3,5
The proposal clearly formulates research hypotheses, rationale, research questions, and aims. It also adequately discusses existing knowledge and literature gaps. The research design is logical and the methods are adequate (fine-tuning a model and multifaceted evaluation of the fine-tuned model). The work plan is clearly described and feasible. The project is innovative as it brings together AI, applied linguistics, clinical practice, and health literacy in a real-world healthcare setting. The project is highly multidisciplinary.	

IMPACT AND DISSEMINATION	3,5
The project will have very high scientific and social impact, as a multidisciplinary project with immediate relevance to healthcare systems. The project will also have very good technological, economical, and environmental impact.	

PRINCIPAL INVESTIGATOR AND RESEARCH ENVIRONMENT	3,5
The CV for the PI is appropriate for the successful execution of the project, as the PI has highly relevant training in applied linguistics and corpus linguistics, experience in working in the medical domain, and a a very good publication record. The mentor and the multidisciplinary team will further help ensure the successful execution of the project and contribute to the career development of the applicant.	

BUDGET AND PROJECT DURATION	3,5
The budget requested is primarily for temporary staff along with a small portion for contractors and dissemination costs. It looks reasonable.	

OVERALL COMMENTS AND SUGGESTIONS	
This is an innovative interdisciplinary project that will have very useful scientific and social impact. My main recommendation would be to further consider how the corpus analysis of linguistic accessibility can be made more systematic in terms of the dimensions analyzed and the specific features for each dimension. While tools like Coh-Metrix may facilitate the analysis, the dimensions that should be analyzed should not be constrained by the tool you use.	

REFEREE 2

SCIENTIFIC QUALITY OF THE PROJECT	4

The scientific background effectively establishes the problem: Hospital Discharge Reports (HDRs) are crucial for post-hospitalization care but are often compromised by complex language, specialized terminology, and inconsistencies, making them difficult for patients, especially those with low health literacy (HL), to comprehend. The proposal cites statistics on inadequate health literacy in Italy to emphasize the local relevance of the problem. It then introduces AI-assisted tools as a promising solution for simplifying medical language and structuring information to align with HL levels, supported by recent studies. The gap identified is the lack of structured research in Italy on integrating AI-generated patient support documents into hospital discharge processes, and the absence of comprehensive evaluation frameworks that incorporate robust linguistic, clinical, and patient-centered measures.
The proposal strongly emphasizes its interdisciplinary nature, bringing together expertise from medicine (nephrologists, cardiologists), AI and computational linguistics, applied linguistics, public health, and legal medicine/bioethics. This collaborative structure aims to ensure scientific rigor, clinical relevance, linguistic accessibility, and responsible innovation throughout the project lifecycle. The team members' roles reflect this, with clinicians contributing to validation, AI and computational linguistics experts co-developing the system, and public health/linguistic researchers leading readability evaluations. The inclusion of legal and bioethical specialists ensures GDPR compliance and ethical oversight.
The proposal is well-structured, clearly defines its objectives, and presents a robust methodology with a strong emphasis on multidisciplinary collaboration and ethical considerations.

IMPACT AND DISSEMINATION	3,5

The project is poised to generate significant scientific impacts by addressing a critical gap in health communication and artificial intelligence integration. The proposal provides novel insights into how AI-driven tools can effectively tailor medical information to different health literacy levels, offering a robust framework for assessing comprehensibility, clinical accuracy, and user satisfaction.

Specifically targeting patients with low health literacy, the project has the potential to mitigate existing health disparities by ensuring equitable access to crucial medical information.
The fine-tuning and validation of generative AI models (Gemma 3 27B, Llama 2 70B) for medical text simplification will push the boundaries of AI applications in healthcare.
The structured evaluation methodologies and potentially the underlying AI frameworks could be adapted and deployed in other clinical settings or for different types of medical documentation.

The proposal outlines a robust and multi-pronged approach to communication and dissemination, ensuring broad reach to both scientific and public audiences. The proposed communication and dissemination actions are high quality, diverse, and well-targeted to reach different stakeholder groups, fulfilling the requirement of identifying at least two actions, one per type.

PRINCIPAL INVESTIGATOR AND RESEARCH ENVIRONMENT	4

The proposal indicates that the applicant, Dr. Pelizzari, possesses the necessary competence, experience, and skills for the successful execution of the project, while also outlining mechanisms for further skill acquisition and professional development. The mentor and team members are positioned to provide substantial added value.

BUDGET AND PROJECT DURATION	3,5

The budget is clear and concise. It is well structured and covers mainly the salary of the PI.

OVERALL COMMENTS AND SUGGESTIONS	
The "AI-mediated discharge document for accessible healthcare (AIM-Health)" proposal presents a strong and well-conceived research plan, with clear objectives and a comprehensive approach to addressing a critical healthcare communication challenge.<br>The proposal clearly articulates its hypothesis, specific research questions, rationale, objectives, and strategic approach. The project aims are well-defined and measurable.<br>The prospective observational study design is logical and well-structured, with a clear phased approach for AI model development, deployment, and evaluation in a real-world clinical setting.<br>The proposed methods are comprehensive, combining quantitative and qualitative measures for clinical accuracy, linguistic accessibility, and patient-perceived utility.	

REFEREE 3

SCIENTIFIC QUALITY OF THE PROJECT	4
The project proposal is of high quality, and it reflects a clearly formulated research objective, research problem, hypothesis, and research steps. The project employs an appropriate and interdisciplinary methodology, is original, and represents high potential from both scientific impact and practical applicability perspectives.	

IMPACT AND DISSEMINATION	4
The project's impact is visible in both significant domains - scientific and societal. It deals with a highly relevant and topical issue, integrates AI in the health sector, and is patient-oriented. The dissemination and communication plan is directed towards both the scientific community, health providers, and patients.	

PRINCIPAL INVESTIGATOR AND RESEARCH ENVIRONMENT	4
The project's PI track records prove her ability to conduct and manage the project, as well as emphasizes her knowledge in the field under scrutiny. The project involves a broad, interdisciplinary team, which combines all knowledge and expertise necessary for a successful management and implementation of the project.	

BUDGET AND PROJECT DURATION	3
The project's budget is presented in large lines, and it may demand more detail to provide a precise overview. It must be noted that the project can be considered an expensive one; nevertheless, the team members' remuneration will be covered from other sources, and this aspect can be evaluated positively. However, the very fact that 77% of the whole budget is reserved for the remuneration of the project's PI and an additional 6,000 euros for the remuneration of the project's auditor can be considered as a proportionally overstretched budgetary position. The position of "other operating expenses" is presented as a final sum and may demand a budgetary breakdown among activities.	

OVERALL COMMENTS AND SUGGESTIONS	
This is a very timely, relevant, well-designed, and well-planned interdisciplinary project.	
